# Cytokine Patterns in COVID-19 Patients: Which Cytokines Predict Mortality and Which Protect Against?

**DOI:** 10.3390/cimb44100323

**Published:** 2022-10-10

**Authors:** Maamoun Basheer, Elias Saad, Majd Kananeh, Layyous Asad, Osama Khayat, Anan Badarne, Zaki Abdo, Nada Arraf, Faris Milhem, Tamara Bassal, Mariana Boulos, Nimer Assy

**Affiliations:** 1 Internal Medicine Department A, Galilee Medical Center, Nahariya 221001, Israel; 2 The Azrieli Faculty of Medicine, Bar-Ilan University, Safad 1311502, Israel; 3 Internal Medicine Department C, Galilee Medical Center, Nahariya 221001, Israel

**Keywords:** lung fibrosis, severity, mortality, cytokines, MMP-7

## Abstract

(1) Background/Aim: People infected with SARS-CoV-2 may develop COVID-19 in a wide range of clinical severity. Pulmonary fibrosis is characterized by several grades of chronic inflammation and collagen deposition in the interalveolar space. SARS-CoV-2 infection has been demonstrated to cause lung fibrosis without a currently elucidated mechanism. Some studies emphasize the role of proinflammatory cytokines. This research studies the correlation of the released cytokines with mortality or lung injury in COVID-19 patients. (2) Methods: Electronic medical record data from 40 patients diagnosed with COVID-19 in the COVID-19 Department, Galilee Medical Center, Nahariya, Israel, were collected. Epidemiological, clinical, laboratory, and imaging variables were analyzed. The cytokine levels were measured upon admission and discharge. A correlation between cytokine levels and severity and mortality or lung involvement was undertaken. (3) Results: IFN-gamma and IL-10 are the most powerful risk factors for mortality in the COVID-19 patient groups in a multivariate analysis. However, in a univariate analysis, TGF-β, CXCL-10, IFN gamma, and IL-7 affected mortality in COVID-19 patients. MMP-7 was significantly correlated with a cytokine storm and a high 4-C (severity) score in COVID-19 patients. MMP-7, TGF-β, IL-10, IL-7, TNF-α, and IL-6 were correlated with high lung involvement in COVID-19 patients. Serum concentrations of IGF-1 were significantly increased upon discharge, but MMP-7 was decreased. (4) Conclusions: Proinflammatory cytokines predict clinical severity, lung fibrosis, and mortality in COVID-19 patients. High concentrations of TGF-β, CXCL-10, IL-10, IL-6, and TNF-α are correlated to severity and lung injury. However, certain cytokines have protective effects and higher levels of these cytokines increase survival levels and lower lung damage. High levels of INF-γ, IL-7, MMP-7, and IGF-1 have protection probabilities against lung injury and severity.

## 1. Introduction

SARS-CoV-2 infection may develop into COVID-19 with a wide range of clinical severity, from a mild upper respiratory tract inflammation to a diffuse viral pneumonia causing acute respiratory failure, including lung injury, multiorgan failure, sepsis, and death [1,2,3]. Acute respiratory distress syndrome (ARDS) is mediated through lung fibrosis, sepsis, septic shock, and acute kidney injury [4,5,6,7,8].

Pulmonary fibrosis is characterized by several grades of chronic inflammation and collagen deposition in the inter-alveolar space [9,10,11]. Lung fibrosis is a well-known complication of ARDS that is histologically characterized by diffuse alveolar damage. Some studies have demonstrated that, while the majority of patients recover without residual lung damage, some patients experience residual fibrotic lesions that are reversible in some cases [10,11]. Several mechanisms have been shown to play a role in the fibrosis cascade and in its progression. However, the pathophysiology of the fibrotic processes is still incompletely defined [10,11,12].

COVID-19 infection has been associated with the super-activation of the host immune system cells. This activation mediates excessive production of proinflammatory cytokines, which may cause tissue injury, particularly lung tissue. The proinflammatory cytokine levels can impact on the clinical performance of the patients [10,11,12,13]. The activated macrophages and neutrophils release profibrotic mediators that promote the accumulation of myofibroblasts [10]. These cells produce an extracellular matrix (ECM) [11,12,13], which establishes a fibrosis loop, and the fibrotic process becomes irreversible [13]. Endothelial injury also causes a release of profibrotic factors and cytokines, such as transforming growth factor–β (TGF-β), which sustains all the fibrotic processes [13,14]. However, the mechanism of fibrosis in COVID-19 disease is still not fully known. Furthermore, there is no explanation for the changes in the cytokine profile upon infection with the virus. The cytokines’ pattern in SARS-CoV-2 infection and their role in the fibrosis and mortality in COVID-19 patients is still not clearly understood.

The purpose of this research is to study the correlation between proinflammatory cytokine levels and the severity of pneumonia, mortality, and lung involvement in COVID-19 patients.

## 2. Materials and Methods

### 2.1. Study Population

Electronic medical record (EMR) data from 40 patients diagnosed with COVID-19 from June 2021 to August 2021, in the Galilee Medical Center’s COVID-19 Department, Nahariya, Israel, were used as the database. The patients were diagnosed based on a positive polymerase chain reaction (PCR) assay for the SARS-CoV-2 virus. None of these patients were vaccinated. It is likely that most of the patients were infected with the Delta strain of the COVID-19 virus.

### 2.2. Study Design

This research is a continuous study to past research (N 231-20) [15]. The analyzed parameters from the EMR data were demographic background, past medical history and treatments, weight, BMI, symptoms before admission (fever, myalgia, dyspnea, and diarrhea), and blood laboratory tests (biochemistry, CBC, blood gases, blood type, coagulation tests, and inflammatory markers). Cytokine storms were defined as described by Caricchio et al. [16]. New predictive criteria were developed, with sensitivity and specificity of 0.85 and 0.80, respectively, comprising three clusters of laboratory results that involve: (1) inflammation, (2) cell death and tissue damage, and (3) pre-renal electrolyte imbalance. The criteria identified patients with longer hospitalization and increased mortality. The 4-C score was calculated as described by Knight et al. [17]. This score contains parameters such as age, sex at birth, number of co-morbidities, respiratory rate on admission, peripheral saturation in room air, Glasgow coma scale, urea, and C-Reactive Protein.

### 2.3. Luminex-Based Multiplex Assay for Serum Cytokine Concentration

Serum cytokine concentrations were measured including CCL-2, CCL-3, CXCL-10, GCSF, IFN-gamma, IL-10, IL-2, IL-4, IL-6, IL-7, TGF-beta, IGF-1, and TNF-α.

Blood samples were withdrawn from moderately ill patients (total of 34) and severely ill patients (total of 6) upon admission and discharge. These samples were kept in a freezer (−30 °C). The samples were incubated for 30 min at room temperature. After coagulation, the blood samples were centrifuged at 1500× *g* at 4 °C for 15 min and the serum was separated and aliquoted into 2 mL tubes and stored in a −80 °C freezer. For cytokine testing, the sera were thawed on ice and pipetted into cryotubes until the assay was done. To assess serum cytokine levels, human high sensitivity cytokine Luminex custom 8-plex kits (R&D Systems, Inc., Minneapolis, MN, USA) were used. Test samples were run in singles, while standard samples were run in duplicates. In brief, color-coded super-paramagnetic beads coated with analyte-specific antibodies were utilized by the Luminex assay. Beads which recognize different target analytes were mixed together and were incubated with the serum sample. Captured analytes were subsequently detected using a cocktail of biotinylated detection antibodies conjugated to streptavidin–phycoerythrin. The magnetic beads were then isolated and measured using the Luminex MAGPIX^®^ Analyzer (R&D Systems, Inc., Minneapolis, MN, USA).

### 2.4. Outcomes

We defined severity upon admission according to the 4-C score. A 4-C score above 10 was considered severe and accounted for a 30% probability of death. We defined critical COVID-19 illness as a composite of admission to the intensive care unit, invasive mechanical ventilation, ARDS, or death. The mortality is most likely to be mediated by lung involvement, hypoxia-mediated arrhythmia, and death.

### 2.5. Ethics

This study was approved by our medical center’s local ethics committee (N 231-20). Retrospective analysis of data from our electronic medical record database was performed under the oversight of the ICH guidelines for good clinical practice.

### 2.6. Statistical Analysis

Statistical analysis was performed using the WinSTAT program. Results are presented as mean + SE for continuous variables. For categorical variables, the frequency and corresponding diagnosis percentage are provided. The Spearman test was used for correlations between two quantitative variables. Univariate direct regression analysis and multivariate stepwise regression analysis were performed for individual variables, including clinical and biochemical variables as an independent variable, and survival or death as the dependent variable. Tests of significance were two-tailed, with a significance level set at less than 0.05. WinSTAT is the statistical add-on program for Microsoft Excel (Kalmia Co., California, MA, USA).

## 3. Results

### 3.1. Clinical Characteristics of the Patients

The patient data are presented in Table 1. The majority of the patients were male and obese. They suffered from hypertension, diabetes mellitus type 2, lung disease (like COPD and IPF), and hemodialysis and had a past medical use of aspirin. The majority of the patients were diagnosed as having severe symptoms upon admission (Table 1). Six patients died (Table 1).

### 3.2. Association between Inflammatory Cytokine Levels and Mortality in COVID-19 Patients

MMP-7, TGF-β, CCL2, CCL-3, CXCL-10, G-CSF, IFN gamma, IL-10, IL-2, IL-4, IL-6, IL-7, TNF-α, IL-6, and IGF-1 were studied for their correlation with mortality in all 40 COVID-19 patients. The results show that TGF-β, CXCL-10, IFN gamma, and IL-7 affect mortality in COVID-19 patients in a univariate regression analysis (Table 2A). Multivariate analysis shows that IFN gamma and IL-10 are the most powerful risk factors for mortality in the COVID-19 patient group (Table 2B). There is a good correlation between IL-10 and INF-γ upon admission, with disease severity and mortality in the COVID-19 patients (Table 2C). The accuracy of the calculations is 96%.

### 3.3. MMP-7 Correlates with Cytokine Storm and 4-C Score Levels

**Figure 1:**
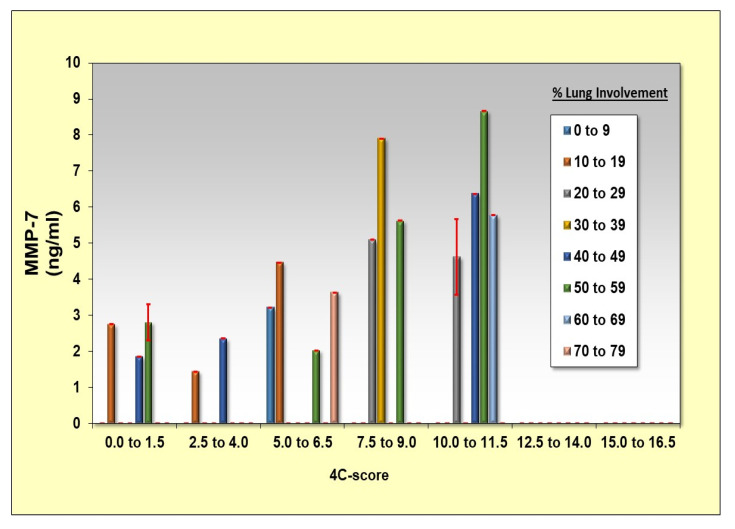
Correlation between MMP-7 concentrations in admission and 4-C score and lung injury (% of consolidation relative to the lung volume involvement).

MMP-7, TGF-β, CCL2, CCL-3, CXCL-10, G-CSF, IFN gamma, IL-10, IL-2, IL-4, IL-6, IL-7, TNF-α, IL-6, and IGF-1 were studied for their correlation to the cytokine storm (as defined by Caricchio, R et al. [15]) and to higher 4-C scores (as detailed by Knight, S.R et al. [16]) in the COVID-19 patient group. The results show that MMP-7 is significantly correlated with the cytokine storm and with a high 4-C score in the COVID-19 patient group (Table 3 and Figure 1).

### 3.4. Association between Inflammatory Cytokine Levels and Lung Involvement (% of Consolidation) in COVID-19 Patients

MMP-7, TGF-β, CCL2, CCL-3, CXCL-10, G-CSF, IFN gamma, IL-10, IL-2, IL-4, IL-6, IL-7, TNF-α, IL-6, and IGF-1 were studied for their correlation to lung involvement in COVID-19 patients. The results show that MMP-7, TGF-β, IL-10, IL-7, TNF-α, and IL-6 were significantly correlated with high lung involvement in COVID-19 patients (Table 4). Il-10 and MMP-7 were shown to have protection properties (Table 4).

### 3.5. The Dynamics in the Cytokine Concentrations in COVID-19 Patients upon Admission and Discharge

**Figure 2:**
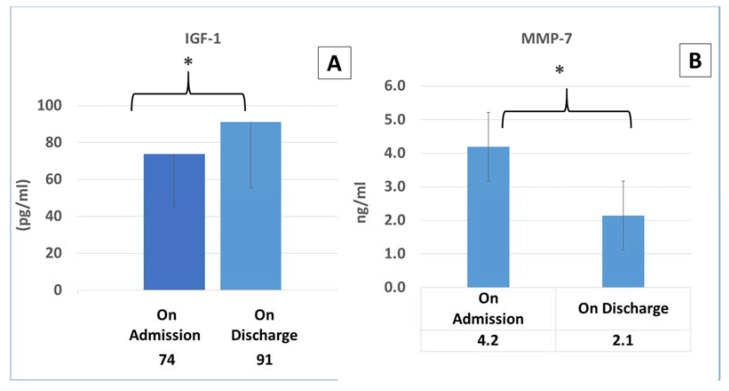
MMP-7 and IGF-1 levels in COVID-19 patients: cytokine levels were measured upon admission and upon discharge. * *p* < 0.05. (**A**,**B**): the dynamics in IGF-1 and MMP-7 upon admission and discharge, respectively.

MMP-7 and IGF-1 cytokine levels were measured upon admission and again upon discharge. IGF-1 significantly increased upon discharge, but MMP-7 decreased as seen in Figure 2.

### 3.6. Correlation between IGF-1 Concentrations in Admission and Lung Injury

**Figure 3:**
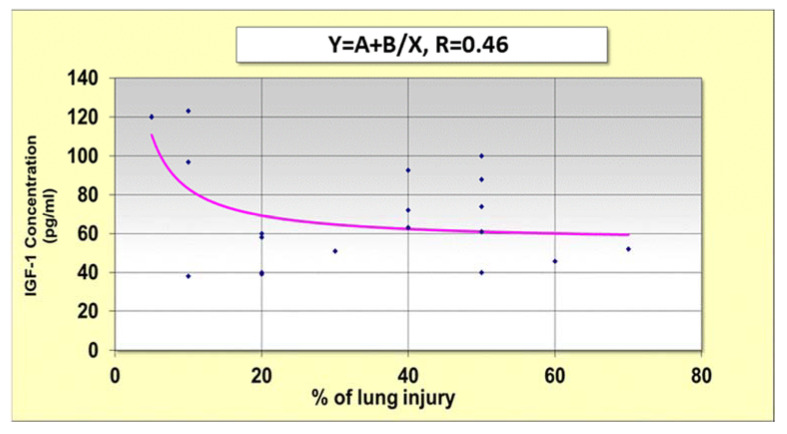
Correlation between IGF-1 concentrations upon admission and % of lung injury.

IGF-1 did not significantly affect the severity or mortality in COVID-19 patients. However, low concentrations of IGF-1 were weakly correlated (R = 0.46) with high lung involvement, and conversely (Figure 3).

### 3.7. Correlation between Cytokine Levels, Gender, and Mortality

**Figure 4:**
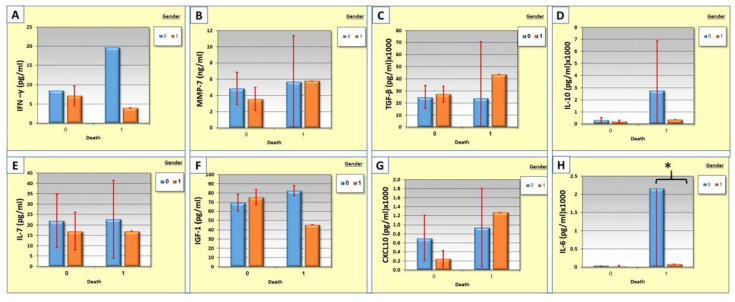
(**A–H**)**:** The correlation between cytokine levels of IFN-γ, MMP-7, TGF-β,IL-10, IL-7, IGF-1, CXCL-10, and IL-6 respectively, gender, and mortality in COVID-19 patients. The blue columns represent males (0) while the orange columns represent females (1).* *p* < 0.05.

The sex of the individuals is associated with a particular type of response. The correlation between cytokine levels, gender, and mortality was measured. Males have high levels of proinflammatory cytokines such as IL-10, IFN-γ, IL-6, IL-7, IL-6, and IGF-1 in the non-survival group (Figure 4). TGF-B and CXCL-10 were higher in female patients in the non-survival group (Figure 4). The differences between the groups are not significant, with the exception of the IL-6 which was significantly higher in the non-survival male group (Figure 4H).

## 4. Discussion

The majority of patients with COVID-19 are asymptomatic or experience mild respiratory illness. Some patients develop a more aggressive disease characterized by fulminant sepsis and acute respiratory failure [17,18,19,20]. The cytokine storm is a critical phase in the deterioration of COVID-19 patients [20].

The purpose of this research was to study the correlation between proinflammatory cytokines and the severity of pneumonia, mortality, and lung involvement in COVID-19 patients. Our results show that IFN-gamma and IL-10 are the most powerful risk factors for mortality in the COVID-19 patient groups in our multivariate analysis (Table 1). However, in univariate analysis, the cytokines TGF-β, CXCL-10, IFN gamma, and IL-7 significantly affected mortality in COVID-19 patients (Table 2).

MMP-7 cytokine levels were correlated with cytokine storm severity and high 4-C scores in the COVID-19 patients (Table 3). MMP-7, TGF-β, IL-10, IL-7, TNF-α, and IL-6 were correlated with high lung involvement in COVID-19 patients (Table 4). Serum concentrations of IGF-1 were significantly increased upon discharge, but MMP-7 levels decreased upon discharge (Figure 2).

When the SARS-CoV-2 virus migrates to the lower respiratory tracts, a secretion of proinflammatory cytokines is released. This mediates the septal terminal fibrosis process, characterized by exacerbated proliferation of fibroblasts and excessive deposition of ECM [17]. M2 macrophages, one of the main immune cells, lead to fibrosis through the secretion of growth-transforming factor-beta (TGF-β). Pulmonary epithelial cell injury and the consequent exposure of the alveolar basal membrane leads to an accumulation of TGF-β1, which induces the recruitment of fibroblasts and ECM production [15,19,20,21,22]. Our results also confirm that high concentrations of TGF-β1 is correlated with severity, lung involvement, and mortality in COVID-19 patients (Table 2 and Table 3).

Interleukin-6 is a cytokine produced during acute and chronic inflammation [23]. Targeting this cytokine is one of the major treatments in treating COVID-19 patients [20,23]. This cytokine induces a transcriptional inflammatory response and is involved in promoting specific differentiation of CD4 naïve T-cells. IL-6 also affects B-cells, T-cells, hepatocytes, hematopoietic progenitor cells, and cells of the central nervous system [23]. Many studies have confirmed the importance of this cytokine in the dangerous cytokine storm phase in COVID-19 patients [2,23,24]. These results align with our results, which show that IL-6 is highly correlated with lung involvement and high mortality rates in COVID-19 patients (Table 2 and Table 3).

High levels of matrix metalloproteinase-7 (MMP-7) have been reported as an inflammatory marker in viral infections [25,26]. MMP-7 is a protease that breaks down the extracellular matrix deposited in the lung after injury [25,26,27]. This cytokine is overexpressed in the lung micro-environment and is increased in the serum of patients with several interstitial lung diseases that may evolve into fibrosis, particularly idiopathic pulmonary fibrosis [27,28,29,30,31]. Our results show that MMP-7 is significantly correlated with the cytokine storm and high 4-C scores in COVID-19 patients (Table 3, Figure 2) and lung involvement, as seen in Table 4. Chun H.J. et al. have shown that MMP-7 levels are significantly increased in patients with severe COVID-19, and these markers could be helpful for distinguishing patients that need invasive mechanical ventilation from those who do not [26]. In our study, MMP-7 levels were higher only in patients that required invasive mechanical ventilation. MMP-7 levels correlated with severity as seen in our results. MMP-7 levels were measured upon admission and again upon discharge. Serum concentrations of MMP-7 significantly decreased upon discharge, as seen in Figure 2. The decrease in MMP-7 levels could be used as a marker of improvement in lung injury, as there is no need for recruiting metalloproteinases to degrade the ECM.

CXCL10 is a powerful recruiter of macrophages [32]. It was recently identified as the chemokine playing a crucial role in COVID-19 [33,34,35]. Elevated serum levels of CXCL10 found in COVID-19 patients are positively correlated with increased disease severity and, more importantly, with an increased risk of mortality [33,34,35,36] as seen in our results above (Table 2 and Table 3).

Interferon-gamma (IFN-γ) is essential for antiviral defense. IFN-γ downregulates viral replication and activates cytokine production by T cells, augmenting the cytotoxic T lymphocyte killing activity [37,38]. However, persistently high levels of IFN-γ worsen systemic inflammation and increase tissue injury and organ failure [39,40]. Nevertheless, persistent low concentrations are problematic in defending against viruses [37,38,39,40]. Our results show that low concentrations of this cytokine are highly correlated with mortality in COVID-19 patients (Table 2).

Interleukin 7 (IL-7) is a cytokine essential for lymphocyte survival and expansion [41]. IL-7 can be safely administered to critically ill COVID-19 patients without exacerbating inflammation or pulmonary injury [41,42,43,44,45,46]. IL-7 is associated with lymphocytes returning to a reference level, appearing to reverse a pathologic hallmark of COVID-19. IL-7 not only restores lymphocyte counts but also reverses T-cell exhaustion [31,32,33,34,35,36,37,38,39,40,41,42,43,44,45,46]. Decreases in IL-7 levels correlated with severity and mortality in critically ill patients, as seen in Table 2, and are highly correlated with lung involvement (Table 4).

One of the important contrasting cytokines is insulin-like growth factor 1 (IGF-1), which has contrasting effects on cell-cycle regulation and proliferation [46]. TGF-β fibrosis is inhibited by IGF-1 (14). IGF-1 could be anti-fibrotic treatment. Our results show that high concentrations of IGF-1 are correlated with low lung involvement (Figure 2). The concentrations of this cytokine were increased upon discharge when patients improved. The recovery in IGF-1 levels is an important phase in lung improvement. Thus, a better understanding of how IGF-I and TGF-β signaling pathways are mutually interconnected is likely to unveil novel targets for the therapeutic intervention of many mediators of lung fibrosis [14].

The uncontrolled production of cytokines, i.e., the cytokine storm, has the strongest link between morbidity and mortality in COVID-19 patients. The cytokine storm is usually a warning sign of COVID-19 escalation and severity, characterized by rapid releases of inflammatory cytokines and chemokines. Table 5 summarizes the cytokines which are protectors against severity and lung injury and cytokines which are predictors of lung injury and severity of COVID-19 patients.

Worldwide COVID-19 epidemiology data indicate differences in disease incidence amongst sex and gender demographic groups. Specifically, male patients are at a higher death risk than female patients [15,46]. Our results also indicate that male patients produce high levels of cytokines, especially the proinflammatory ones (Figure 4). Tianyuan Liu et al. also showed that men exhibit higher levels of proinflammatory cytokines. These data suggest the existence of different basal immunophenotypes amongst different demographic groups, which are relevant to COVID-19 progression and may contribute to explaining sex biases in disease severity [46].

## 5. Limitations

The sample size in this study is small and the association between clinical characteristics and cytokine levels was not evaluated in the current study. Thirty-four patients survived the disease versus six who did not. Follow-up was also not performed, as it would be interesting to evaluate the changes of cytokines according to days after discharge from the hospital. A large sample size should be used in order to emphasize the results. However, even with 40 patients, our study demonstrates the pattern of cytokines in COVID-19 patients.

## 6. Conclusions

Proinflammatory cytokines predict clinical severity, lung fibrosis and mortality in COVID-19 patients. The uncontrolled production of cytokines, the cytokine storm, has links between morbidity and mortality in COVID-19 patients. However, other cytokines have protective effects and higher levels of these cytokines increase the likelihood of survival.

## Figures and Tables

**Table 1:** Clinical characteristics of the surviving and non-surviving patients with COVID-19 infection.

VARIABLE	
***Total***	***N* = 40**
Age	63 ± 18
Male (%)	56%
BMI	29.4 ± 6
**Comorbidities %**	
Diabetes (%)	42
Hypertension (%)	53
Lung disease (%)	7
Hemodialysis (%)	0
Aspirin use (%)	50
**Symptom’s duration before admission to hospitals** **(days)**	6 ± 4
**Symptoms before admission** **(% of total)**	
Fever %	61
Diarrhea %	0
Dyspnea %	58
Clinical severity upon admission %	23
**Lab Findings upon admission**	
Hemoglobin (mg/dL)	12.5 ± 1.5
Absolute neutrophil count (×10^3^/µL)	4.4 ± 2.6
Absolute lymphocyte count (×10^3^/µL)	1.07 ± 0.7
Neutrophil to lymphocyte ratio (NLR)	5.7 ± 4.7
Platelet (×10^3^/µL)	190 ± 92
BUN (mg/dL)	17.5 ± 8.8
Creatinine (mg/dL)	1.01 ± 1.0
Triglycerides (mg/dL)	140 ± 57
HDL (mg/dL)	28 ± 10
C-reactive protein (CRP) (mg/dL)	99 ± 86
Ferritin (µg/L	466 ± 325
D-dimer (ng/mL)	1080 ± 813
Fibrinogen (mg/dl)	677 ± 165
ALT (U/L)	24 ± 16
4-C score	7.0 ± 4.5
O_2_ supplement upon admission %	11
High flow use (% of total)	11
Survival (% of total)	85

Abbreviation: BMI: Body mass index, ALT: Alanine transaminase.

**Table 2:** Correlations between inflammatory cytokine levels upon admission and mortality in COVID-19 patients. The cytokines are: MMP-7, TGF-β, CCL2, CCL-3, CXCL-10, G-CSF, IFN gamma, IL-10, IL-2, IL-4, IL-6, IL-7, TNF-α, IL-6, and IGF-1. **A**: Univariate analysis of the strength of cytokine with mortality prediction. **B**: Multivariate analysis of the strength of cytokine levels with mortality prediction. SE are the standard errors of the regression coefficients. T is the quotient of the coefficient. Two-sided *p* values or observed significance levels. **C**: The validity (predictive power) of IL-10 and INF-γ upon mortality in COVID-19 patients. The accuracy of the calculations is 96%.

A.					
	Coefficient	95% Conf. (±)	Std. Error	T	*p*-Value
***Constant***					
MMP-7	−0.02	0.05	0.02	−0.81	0.44
**TGF-β**	**2.4**	**0.0001**	**7.19**	**3.34**	**0.01**
CCL2	0.0002	0.0005	0.00002	−1.06	0.32
CCL3	0.01	0.018	0.007	1.9	0.1
**CXCL-10**	**0.000**	**0.00**	**0.0001**	**3.81**	**0.008**
G-CSF	0.002	0.05	0.002	1.22	0.26
**IFN gamma**	**−** **1.85**	**1.95**	**0.79**	**−** **2.32**	**0.049**
IL-10	−0.008	0.001	0.0005	−1.64	0.15
IL-2	-0.01	0.03	0.01	−0.761	0.47
IL-4	5.67	5.89	2.4	2.35	0.056
IL-6	0.002	0.006	0.002	0.99	0.358
**IL-7**	**−** **0.02**	**0.02**	**0.008**	**−** **2.44**	**0.04**
TNF-α	−0.005	0.01	0.007	−0.67	0.52
IL-6	0.004	0.006	0.002	1.7	0.13
IGF-1	0.001	0.004	0.001	0.688	0.51
**B.**					
	**Coefficient**	**95% Conf. (±)**	**Std. Error**	**T**	***p*** **-Value**
***Constant***					
IFN gamma	0.07	0.048	0.023	−2.98	0.007
IL-10	0.0005	0.0002	0.0001	3.87	0.001
**C.**					
		**Actual Count**		**0**	**1**
**Died patients**		3		3	0
**Survived patients**		22		1	21

**Table 3:** The MMP-7 cytokine level upon admission is correlated with the cytokine storm (**A**) and with a higher 4-C scores (**B**), respectively. SE are the standard errors of the regression coefficients. T is the quotient of the coefficient. Two-sided p values or observed significance levels.

A.					
	Coefficient	95% Conf. (±)	Std. Error	T	*p*-Value
***Constant***					
**MMP-7**	**0.097**	**0.084**	**0.04**	**2.42**	**0.025**
**B.**					
	**Coefficient**	**95% Conf. (±)**	**Std. Error**	**T**	***p*** **-Value**
***Constant***					
**MMP-7**	**1.48**	**0.56**	**0.27**	**5.44**	**0.00002**

**Table 4:** Association between inflammatory cytokine levels upon admission and lung involvement (% of consolidation) in COVID-19 patients. SE are the standard errors of the regression coefficients. T is the quotient of the coefficient. Two-sided *p* values or observed significance levels.

	Coefficient	95% Conf. (±)	Std. Error	T	*p*-Value
***Constant***					
MMP-7	−18.027	8.5	0.38	−4.7	0.0008
TGF-β	0.001	0.0008	0.0003	2.7	0.022
IL-10	−0.04	0.042	0.019	−2.45	0.033
IL-7	1.5	0.93	0.41	3.56	0.005
TNF-α	2.01	1.52	0.686	2.9	0.014
IL-6	0.97	0.44	0.199	4.8	0.0006

**Table 5:** Summary of cytokines which are protectors against severity and lung injury, and cytokines which are predictors of lung injury and severity of COVID-19 patients. High concentrations of TGF-β, CXCL-10, IL-10, IL-6, and TNF-α are correlated with severity and lung injury. On the other hand, high levels of INF-γ, IL-7, MMP-7, and IGF-1 have protection probabilities against lung injury and severity.

Protectors	Predictors
IFN-γ	TGF-β
IL-7	CXCL-10
MMP-7	IL-10
IGF-1	IL-6
	TNF-α

## Data Availability

The data presented in this study are available on request from the corresponding author. The data are not publicly available due to privacy of the patient.
